# Breast cancer awareness among older women

**DOI:** 10.1038/sj.bjc.6604668

**Published:** 2008-09-23

**Authors:** L Linsell, C C Burgess, A J Ramirez

**Affiliations:** 1 Kings College London, Cancer Research UK London Psychosocial Group, Institute of Psychiatry, St Thomas’ Hospital, London SE1 7EH, UK

**Keywords:** breast cancer, older women, cancer awareness

## Abstract

The aim of this study was to elicit the level of breast cancer awareness in older women. A cross-sectional study-specific questionnaire survey of 712 British women aged 67–73 years (response rate 83.8%), assessing knowledge of symptoms and risk and confidence to detect a change, was conducted. Over 85% of respondents were aware that a lump was a symptom of breast cancer but knowledge of non-lump symptoms was limited. Knowledge of risk was poor; 50% believed that the lifetime risk of developing breast cancer was less than 1 in 100 women and 75% were not aware that age is a risk factor. Thirty-one percent of women reported low levels of confidence to detect a breast change and 19% rarely or never checked their breasts. Those with fewer educational qualifications had poorer knowledge of symptoms, less awareness of lifetime and age-related risks, but were more likely to check their breasts than more highly educated women. This national survey demonstrates a significant lack of the prerequisite knowledge and confidence to detect a breast change. Raising breast cancer awareness and promoting early presentation among older women is important, as they are more at risk of breast cancer and more likely to delay seeking help with breast cancer symptoms than younger women.

Breast cancer is predominantly a disease of older women. Most breast cancers occur in older women (McPherson *et al*, 2000), with approximately one third of all breast cancers occurring in women over the age of 70 (Office for National Statistics, 2005). Furthermore, older women are more likely to delay their presentation with breast cancer (Ramirez *et al*, 1999). This is of concern given that delay in the presentation of breast cancer of 3 months or more result in diagnosis with later stage disease and reduced chances of survival (Richards *et al*, 1999). Survival from breast cancer is in fact worse among women over 70 years compared with younger women. The relative 5-year survival rates from breast cancer are 80% for women aged less than 70 years, 66% for those aged 70–79 years and 47% for those aged 80–89 years (McPherson *et al*, 2000).

The NHS Breast Screening Programme (NHSBSP) has an upper age limit, which leaves older women unprotected by routine mammographic screening. At the same time, among older women there is a high chance that a breast symptom is one of breast cancer. Over 1 in 3 breast symptoms in older women (⩾65) are because of cancer, whereas about 1 in 10 breast symptoms are malignant in women under 65 years (Nichols *et al*, 1980). Older women need to be equipped with the relevant knowledge and confidence to detect and seek medical help promptly for a breast change.

The aims of this national survey were to: (1) describe the levels of knowledge of breast cancer symptoms and risk in older women, and assess their confidence to detect a breast change; (2) examine levels of knowledge and beliefs in relation to sociodemographic characteristics to determine which older women are most at risk of delayed presentation. The ultimate aim of the survey is to inform interventions that promote early help seeking and increase survival in older women with breast cancer.

## Materials and methods

### Participants

The survey sample comprised 850 women aged 67–73 years, an age group leaving the routine protection of the NHSBSP. Age Concern Research Services conducted sampling and data collection through their 50plusview panel. This panel consists of 10 000 people aged 50+ years, recruited in two ways: 60% from existing panels recruited by a private sector organisation, GfK information services, and 40% through a recruitment campaign among Age Concern donors. GfK recruit using random digit dialling telephone sampling and aim to fill quotas designed to achieve a representative sample of the UK population, based on age, sex, region and other sociodemographic characteristics. Age Concern recruit their members using an annual donor mailing advertising voluntary membership to the Panel. We invited every woman aged 67–73 years from the panel to participate. We examined the representativeness of the respondents by comparing the sociodemographic characteristics of women recruited to the survey to those of the general female population in the United Kingdom using estimates published by the Office of National Statistics.

### Questionnaire

A letter outlining the survey together with a study-specific questionnaire was sent to each woman in November 2005. The questionnaire was piloted on older women in breast screening clinics and examined the following areas: knowledge of breast cancer symptoms, knowledge of the risk of developing breast cancer and confidence to detect breast changes. In the first question, participants were provided with a description of 11 breast cancer symptoms, taken from public education leaflets (Austoker, 1991) and asked which ones were symptoms of breast cancer. A score was produced of the number of symptoms correctly identified (range 0–11). Details of the other categorical items used in the questionnaire are shown in Table 2. Sociodemographic data (marital status, educational qualifications and ethnic group) were also collected and the Townsend Index of Multiple Deprivation (IMD) of ward of residence was calculated from the participant's postcode (ODPM Publications, 2004). This is a measure of socioeconomic status with a higher score (range 1–100) indicating greater deprivation.

### Statistical analysis

Summary statistics were reported for all the main questionnaire items. The association between knowledge of symptoms and each sociodemographic variable was firstly examined using the Wilcoxon rank-sum test (for comparison of two groups) and Kruskal–Wallis test (for comparison of more than two groups), followed by a multivariate analysis using ordinal logistic regression. The knowledge of risk items and breast checking were collapsed into binary variables and bivariate analyses were performed using Pearson's χ^2^ test (for comparison of two groups) and the non-parametric test for trend (for comparison of more than two ordered groups). This was followed by a multivariate analysis using logistic regression. For all outcomes, sociodemographic variables with a *P*-value<0.2 in the bivariate analyses were entered into the multivariate model, then retained in the final model if *P*<0.05. All analyses were conducted using Stata version 9.1.

## Results

Of the 850 participants surveyed, 712 completed and returned the questionnaire, a response rate of 83.8%. The sociodemographic characteristics of the sample compared with the general population of older women (where possible) are shown in Table 1. Geographical region, ethnicity and retirement status were broadly similar, however, the women appeared to be more affluent and highly educated compared with the general population of women this age. Also, a higher proportion of women in the sample reported to be living alone.

### Knowledge of breast cancer symptoms

**Figure 1:**
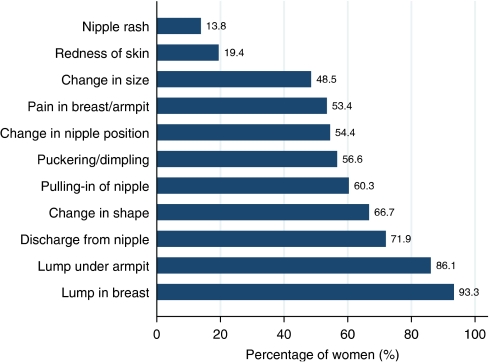
Proportion of older women identifying each breast symptom from a set of 11 potential breast cancer symptoms (*n*=712).

Most women (over 85%) were aware that a lump in the breast or under the armpit was a symptom of breast cancer (Figure 1). However, they were less knowledgeable about non-lump symptoms; less than half of the women recognised a change in size, redness of skin and nipple rash as signs of breast cancer. The median number of symptoms identified from the set of 11 provided was 6 (IQR: 4–9) for the whole sample (Mean: 6.2, 95% CI: 6.0–6.5). Knowledge did not vary by ethnic group, social deprivation or relationship status, however, it was associated with education (Table 3). Women with an O level qualification or above identified a median of seven symptoms (IQR: 5–9) compared with a median of five symptoms (IQR: 3–8) in women with no educational qualifications and were just over twice as likely to identify at least one additional symptom (*P*<0.001).

### Knowledge of risk of developing breast cancer

Women were overly optimistic regarding a woman's lifetime risk of developing breast cancer, with half believing that the chances were less than 1 in 100 (Table 2). Around a third (36.7, 95% CI: 33.1–40.5%) correctly indicated a 1 in 9 risk, whereas a small proportion (13.3%) overestimated the risk to be 1 in 3. Women with a higher level of education were more likely to estimate lifetime risk correctly, but there was no association with the other sociodemographic variables (Table 3). This remained significant only for the most qualified group (A levels/degree or above) in the logistic regression analysis; the odds of responding correctly was 1.65 times higher in this group compared with women with no qualifications (*P*=0.019).

Most of the women were also not aware of the increased risk of breast cancer with age; only a quarter (25.1, 95% CI: 21.9–28.5%) correctly believed they were more at risk whereas 61.7% perceived no difference (Table 2). Awareness of this risk was strongly associated with higher levels of education but not associated with any other sociodemographic characteristics (Table 3). The odds of responding correctly was 2.24 times higher in women with ‘O’ level qualifications and 3.4 times higher in the most qualified group compared with women with no qualifications (*P*<0.001). Interestingly, among those who gave the incorrect answer, the most educated group appeared more likely to believe that old age decreases the likelihood of the disease (24.4% (21/86) *vs* 14.9% (56/377) in the two lower groups combined).

### Confidence to detect breast changes

Around two thirds of the women reported that they checked their breasts on a weekly or monthly basis (65.9, 95% CI: 62.3–69.4%), but one fifth claimed that they rarely or never checked their breasts for changes (Table 2). Furthermore, nearly one third of women were not confident that they would be able to detect a breast change and 15% were not confident about how their breasts normally look and feel. There were no associations between breast checking and the sociodemographic variables apart from education (Table 3). More highly educated women were less likely to check their breasts compared with women with no qualifications, but this was only significant for the most highly educated group (*P*=0.024).

## Discussion

The results of the survey indicate that although older women demonstrate some knowledge of the symptoms and risks associated with breast cancer, there was poor awareness about important issues, particularly among those who were less educated. The main insufficiency in knowledge was the lack of recognition of non-lump symptoms as symptoms of breast cancer and a poor understanding about risk. Around half of the women regarded their lifetime risk of developing breast cancer as less than 1 in 100, and the majority (approximately 75%) believed they had the same or lower risk than younger women. There were also a substantial number of older women who rarely or never checked their breasts and had a lack of confidence in being able to detect a breast change.

Our findings are in line with other research; large-scale, population-based surveys across a broad range on cancer types in the United Kingdom have consistently shown poor knowledge of the early warning signs of cancer (Brunswick *et al*, 2001; Grunfeld *et al*, 2002; McCaffery *et al*, 2003) and limited awareness of risk factors (Wardle *et al*, 2001; Grunfeld *et al*, 2002; McCaffery *et al*, 2003; Waller *et al*, 2004b; Robb *et al*, 2006). However, cancer awareness among older age groups has been shown to be even poorer (Grunfeld *et al*, 2002; Adlard and Hume, 2003; Waller *et al*, 2004b; Webster and Austoker, 2006). Of equal concern is the lack of knowledge that age is a risk factor for breast cancer, as well as some other types of cancer (Grunfeld *et al*, 2002; McCaffery *et al*, 2003; Waller *et al*, 2004a; Moser *et al*, 2007). A large national survey (Moser *et al*, 2007) found that only 1% of women (all ages) were correctly informed that a woman is most likely to get breast cancer in her 80s. Furthermore, Grunfeld *et al* (2002) found that older women actually perceived a reduced personal risk compared with their younger counterparts.

Existing research also suggests that cancer awareness is poorer among those who are less well educated and those with a lower socioeconomic status (Brunswick *et al*, 2001; Wardle *et al*, 2001). We demonstrated that knowledge of symptoms and risk were strongly related to educational qualifications, a similar trend was found in the Grunfeld survey (Grunfeld *et al*, 2002) with socioeconomic status. Webster and Austoker (2006) reported an association between inaccurate knowledge of lifetime risk of breast cancer and lower formal education, and McCaffery *et al* (2003) found lower levels of knowledge about bowel cancer in less educated groups.

The strength of this national survey relates to the uniquely large sample of older women in the United Kingdom on which it is based. However, although we concentrated on an older age group, the survey participants had a relatively young age distribution (67–73 years) and the most elderly were not represented. Also, the sample participants in our survey were wealthier and more highly educated than the general population of women in this age group. Responders to surveys are frequently of higher socioeconomic status and education than non-responders (Purdie *et al*, 2002; Martikainen *et al*, 2007) and the sampling methods used for recruitment to the 50plusview panel may have contributed to this, in particular, the Age Concern sample, which was recruited from donors to the charity. There may be other, unknown and unmeasured, psychosocial characteristics associated with being a charity donor that may also be associated with breast cancer awareness; these could also bias our estimates of breast cancer awareness. Overall, our findings are likely to present a more positive picture of knowledge and attitudes towards breast cancer than is the case among all older women nationally and, therefore, overestimate levels of breast cancer awareness in older women. Higher socioeconomic status, education and other unmeasured characteristics may explain why the older women in this sample were more breast cancer aware compared with the women of the same age in the Grunfeld survey, which was part of an ONS Omnibus survey (Grunfeld *et al*, 2002).

If cancer awareness among the general public is limited then people are ill-equipped to make informed decisions about their health, which may consequently lead to delayed presentation and poorer survival (Ramirez *et al*, 1999; Richards *et al*, 1999; Coleman *et al*, 2003; MacDonald *et al*, 2006). A number of initiatives and programmes aiming to raise risk and symptom awareness and promote early presentation are currently underway, such as the Open up to Mouth Cancer campaign (Cancer Research UK, 2005), Touch Look Check (TLC) campaign (Breakthrough Breast Cancer) and the SunSmart campaign (Cancer Research UK, 2003). The Department of Health is investing in a programme that uses social marketing techniques to raise awareness of the signs and symptoms of breast, lung and bowel cancers and to encourage people, who think they might have cancer, to seek help sooner. A psycho-educational intervention delivered by health professionals to promote the earlier presentation of breast cancer in older women is also currently being developed and tested (Burgess *et al*, 2008; Burgess *et al* (stage II), submitted). Such initiatives are supported by the NHS Cancer Reform Strategy (Department of Health, 2007), along with the development of a measurement tool for symptom and risk factor awareness across all cancer types. This tool will enable researchers to monitor changes in cancer awareness over time and evaluate the impact of interventions. For such interventions to be successful in tackling late diagnosis and improving cancer outcomes, they rely on a strong body of research to provide information on whom to target, and how to target most effectively.

## Figures and Tables

**Table 1:** Sociodemographic characteristics of older women (*n*=712)

	**Percentage (*n*) of sample**	**Percentage of UK population**	**Source of data for UK population estimates**
*Geographical region (*n*=705)*			
The North	22.4 (158)	25.1	Mid-2004 UK population estimates for females aged 65–74 years, Office of National Statistics (ONS)
Midlands	16.3 (115)	16.2	
London	10.6 (75)	9.2	
South and eastern	24.7 (174)	22.9	
Southwest	10.9 (77)	9.3	
Wales	5.5 (39)	5.3	
Scotland	8.5 (60)	9.3	
Northern Ireland	1.0 (7)	2.6	
			
*Ethnic background (*n*=711)*			
White-British	89.2 (634)	92.5	Females aged 65–74 years in England and Wales, 2003 (ONS)
Pakistani	6.2 (44)		
Black-Caribbean	2.0 (14)		
Other minority	2.6 (19)		
			
*Education (*n*=694)*			
No qualifications	42.8 (297)	58	Females aged 65–74 years, Focus on older people 2005 (UK), Family resources survey (ONS)
Up to O level/school certificate	26.4 (183)		
Up to A level/higher certificate	9.9 (69)		
Degree or above	10.2 (71)		
Other	10.7 (74)		
			
Retired (*n*=633)	90.8 (575)	88	Females aged 65–74 years, Focus on older people 2005 (UK), Family resources survey (ONS)
			
*Marital status (*n*=708)*			
Married/cohabiting	54.4 (385)	60	Mid-2004 UK population estimates for females aged 65–74 years (ONS)
Widowed	29.7 (210)	26	
Single	6.5 (46)	4	
Divorced/separated	9.5 (67)	9	
			
Living alone (*n*=633)	43.9 (278)	33	Mid-2004 UK population estimates for females aged 65–74 years (ONS)
			
*Type of tenure (*n*=630)*			
Owner occupied	88.7 (559)	73	By reference person aged 65–84 years: Focus on older people 2005
Rented – social	9.0 (57)	24	(UK), General Household survey (ONS)
Rented – private	2.2 (14)	4	
*Number of cars (*n*=626)*			
None	29.1 (182)	27	All UK households 2004, ONS
1 car	57.5 (360)	45	
2 or more cars	13.4 (84)	29	
			
*Townsend Index of Multiple Deprivation (IMD)*^a^ *(*n*=610)*			The English Indices of Deprivation 2004, April 2004, ODPM
Median (Interquartile range)	13.4 (8.2–21.5)	16.7 (9.7–30.2)	Publications

a IMD scored 1 to 100. Higher scores indicate greater deprivation.

**Table 2:** Summary of responses to breast cancer awareness questionnaire (*n*=712)

**Questionnaire item**	**Response categories**	**Median (IQR)**
*Knowledge of breast cancer symptoms*		
Which of the problems below do you think could be a symptom of breast cancer? (*n*=712)	Scattered list of 11 breast cancer symptoms provided	6 (4–9)
		
		***n* (%) of women**
*Knowledge of risk*		
How many women will develop breast cancer in their lifetime? (*n*=686)	1 in 3 women	91 (13.3)
	1 in 9 women	252 (36.7)
	1 in 100 women	215 (31.3)
	1 in 1000 women	109 (15.9)
	1 in 10 000 women	19 (2.8)
		
Does your age make you more or less likely to develop breast cancer? (*n*=705)	More likely	177 (25.1)
	No difference	435 (61.7)
	Less likely	93 (13.2)
		
*Confidence to detect breast changes*		
How often do you examine your breasts (*n*=707)	Weekly	193 (27.3)
	Monthly	273 (38.6)
	Every 6 months	104 (14.7)
	Rarely or never	137 (19.4)
		
How confident are you that you would notice a change in your own breasts? (*n*=708)	Very much	107 (15.1)
	Quite	381 (53.8)
	A bit/not at all	220 (31.1)
		
To what extent do you know how your breasts normally look and feel? (*n*=704)	Very much	295 (41.9)
	Quite	301 (42.8)
	A bit/not at all	108 (15.3)

**Table 3:** Knowledge of symptoms and risk, and confidence for women with different educational qualifications

**Knowledge of symptoms: number of breast symptoms identified**					
**Education**	***n***	**Mean (s.d.)**	**Median (IQR)**	**Odds Ratio (95% CI)**	***P*-value**
No qualifications	297	5.5 (2.9)	5 (3–8)	1.00 (referent)	
To O level/school certificate	183	6.8 (2.7)	7 (5–9)	2.16 (1.56–2.99)	<0.001
To A level/Degree or above	140	6.8 (2.7)	7 (5–9)	2.22 (1.56–3.16)	<0.001
					
**Knowledge of risk: how many women will develop breast cancer in their lifetime?**					
**Education**	***n***	**Number (%) with correct response (1 in 9)**		**Odds Ratio (95% CI)**	***P*-value**
No qualifications	284	91 (32.0)		1.00 (referent)	
To O level/school certificate	179	68 (38.0)		1.30 (0.88–1.92)	0.19
To A level/Degree or above	137	60 (43.8)		1.65 (1.09–2.51)	0.019
					
**Knowledge of risk: does your age make you more or less likely to develop breast cancer?**					
**Education**	***n***	**Number (%) with correct response (more likely)**		**Odds Ratio (95% CI)**	***P*-value**
No qualifications	295	46 (15.6)		1.00 (referent)	
To O level/school certificate	181	53 (29.3)		2.24 (1.43–3.51)	<0.001
To A level/Degree or above	140	54 (38.6)		3.40 (2.14–5.40)	<0.001
					
**Confidence: how often do you examine your breasts?**					
**Education**	***n***	**Number (%) very or quite confident**		**Odds Ratio (95% CI)**	***P*–value**
No qualifications	296	206 (69.6)		1.00 (referent)	
To O level/school certificate	180	118 (65.6)		0.83 (0.56–1.23)	0.36
To A level/Degree or above	140	82 (58.6)		0.62 (0.41–0.94)	0.024
